# What You Can Do: A Qualitative Study on Black Maternal Mental Health and Equity

**DOI:** 10.3390/healthcare14010061

**Published:** 2025-12-26

**Authors:** Amittia Parker

**Affiliations:** Thrive Center for Children, Families, and Communities, Georgetown University, Washington, DC 20007, USA; amittia.parker@georgetown.edu; Tel.: +1-202-784-3477

**Keywords:** Black maternal mental health, health equity, qualitative research, perinatal mental health, healthcare disparities, social support

## Abstract

**Background/Objectives**: Maternal mental health concerns are a leading cause of maternal morbidity and mortality, disproportionately impacting Black mothers in the United States. Structural racism and social determinants of health contribute to increased risks of perinatal mental health issues, limited access to formal services, and adverse health outcomes for Black mothers. While formal mental health services are underutilized, Black mothers employ a variety of culturally relevant and context-specific strategies to support their mental health. This study seeks to understand the barriers, preferences, and experiences that guide their decision-making and inform culturally responsive care. **Methods**: This qualitative study employed thematic analysis of in-depth interviews conducted with 12 Black mothers aged 20–39 residing in a midwestern metropolitan area. The research explored individual experiences, preferences for support, and perspectives on healthcare to identify pathways for advancing mental health equity. **Results**: Three major themes emerged: (1) Expanding conceptions of mental health support beyond traditional services, emphasizing preferences for culturally congruent, convenient, and stress-decreasing interventions; (2) The salience of past experiences and identities in shaping support preferences and decisions; (3) What healthcare professionals can do, the knowledge and skills healthcare professionals can gain, and the actions that they can to become more helpful to Black mothers. The importance of healthcare professionals embodying nonjudgmental, patient, and caring attributes, as well as strengths-based, culturally responsive approaches in care. **Conclusions**: Advancing mental health equity for Black mothers requires increased awareness of existing disparities, barriers to care, and the strengths embedded within their communities. This research provides actionable insights for healthcare providers, policy makers, and researchers to identify, assess, and respond to the unique needs of Black mothers through culturally responsive and participatory approaches. Findings have implications for intervention design, theory development, and policy reform to improve mental health outcomes.

## 1. Introduction

Maternal mental health equity is needed now. Everyone does not have access to high-quality health and mental health care, and positive experiences within systems of care, regardless of race, ethnicity, gender, socioeconomic status, sexual orientation, or geographic location [1,2,3,4]. Examining what happened, what is happening, and listening to and being guided by those most impacted by mental health inequities is the pathway towards achieving maternal mental health equity [5].

Maternal or perinatal mental health concerns are a leading cause and complication in the maternal mortality crisis. The leading causes of maternal mental health-related deaths are homicide, suicide, and overdose [6,7]. Even when considering the maternal mental health-related causes, serious illness, and other obstetric conditions, 4 out of 5 maternal deaths during the perinatal period from pregnancy and the postpartum period up to a year after birth are preventable [8]. Additionally, it is important to note that the risk for maternal mortality and near deaths is present through the entire perinatal period; most of the deaths occur from day 42 through 1 year after birth [8]. This means that mothers are dying and leaving behind very young children and families, and it is an early adversity that is traumatic and preventable.

Understanding when the maternal deaths are occurring and who is most at risk of experiencing mortality, near-death experiences, and complications is an important development in solution-building to eliminate the maternal mortality crisis. Black mothers experience persistent racial maternal health and mental health disparities in the United States and across the globe [9]. Black mothers are more likely to die than any other racial group in the United States (50.3 deaths per 100,000 live births) [10]. Also, Black mothers residing in Southern states within the United States are among the most likely to experience a perinatal fatality [10]. This is due to Black mothers consistently facing unique, multidetermined risks, including gendered racism, social and economic adversity, and limited access to culturally responsive mental health care [9,11,12]. This population is disproportionately affected by poorer maternal health outcomes, including higher rates of maternal morbidity and maternal mortality. Maternal mental health concerns are a leading cause and complication within the maternal mortality crisis. Suicide, homicide, and overdose are leading maternal mental health-related death causes, and considered together, the overwhelming majority of maternal deaths have been deemed preventable [13]. This is unacceptable.

The intersectionality of race, gender, and mothering leaves Black mothers subject to distinctive stressors and barriers, which not only undermine mental health but also perpetuate cumulative disadvantage that impacts the mother, their young child, and family in lasting ways [14,15]. At the same time, Black mothers experience unique strengths, and more attention towards the strengths, solutions, or appropriate ways to intervene is needed [5,16]. Closing the gaps and advancing mental health equity is an urgent imperative for today and future generations that requires a culturally responsive, community-driven approach that centers the needs, preferences, and experiences of Black mothers and those who care for them. The objective of this paper is to address a gap in the literature and in practice by illuminating how Black mothers navigate supports for maternal mental health (e.g., informal, community, and formal health/mental health), and also to highlight the ways that healthcare professionals can advance Black maternal mental health, Black autonomy and mental health decision-making, and access to services and supports.

## 2. Literature Review

### 2.1. Maternal Mental Health Disparities and Determinants

Decades of research highlight disproportionately adverse outcomes for Black mothers in the United States, including chronic health conditions, maternal morbidity or near deaths, and maternal mortality. Black mothers are 3 times more likely to die during the perinatal period, and also 2–3 times more likely to experience miscarriage, stillbirth, and infant mortality than white mothers [13,17]. These disparities are rooted in both the historical legacy of structural racism and the present-day social determinants of health, including poverty, housing insecurity, exposure to violence, and economic instability [12]. Black mothers are also at elevated risk for severe, chronic, and debilitating mental health challenges compared to their White peers [18,19]. Given that maternal mental health challenges are experienced in the context of cumulatively more negative social and economic conditions, the impacts can be lasting and impact generations, more specifically, the consequences extend to adverse birth outcomes such as low birth weight, prematurity, and mortality, parent–child relational challenges, and the growth and development of the child [20].

### 2.2. Barriers to Formal Mental Health Services

While increasing numbers of Black women and mothers are engaging in formal mental health care services, issues of access, barriers, and negative experiences in care persist [21]. Barriers to formal mental health services for Black mothers span from individual-level factors—such as stigma and spiritual beliefs—to structural challenges including provider supply, insurance coverage, and discriminatory practices [21,22,23]. Cultural perceptions that reinforce privacy or equate help-seeking with weakness, such as the superwoman complex [24], continue to restrict engagement with formal services [25]. Additionally, lower perceived quality of care, logistical challenges, and experiences of intercultural disconnect appear to amplify mistrust and use of formal supports [26,27,28]. Further, the perception of racism and experiences with discrimination deeply shape maternal mental health by way of racialized stress, mental health symptoms, and the willingness to access services [1]. It is apparent that various structural factors, including racism, create “fractures in health care systems that create barriers for accessing care and lack of shared decision making disempowers birthing people from fully and authentically actualizing their needs and desires.” [29].

### 2.3. Informal and Community Supports

Research increasingly recognizes that Black mothers prefer and rely upon informal supports (family, friends) and community-based resources (faith groups, workplace supports, childcare providers) to address mental health needs, often concurrently. These supports offer culturally congruent, accessible, and empowering interventions for emotional regulation, practical assistance, and social companionship [30,31]. Informal supports are often perceived as safer and more effective than formal services, particularly when trust and confidentiality are preserved [32,33]. Conversely, studies have highlighted how breaches in trust or negative experiences drive preferences for self-reliance or increased autonomy in decision-making about supports [34]. Community resources, such as doulas, support groups, spiritually oriented spaces (churches, mosques, centers), and Black-led organizations (e.g., beauty salons, associations, Greek organizations, workshops), play an essential role in promoting resilience, coping, and well-being for Black mothers [29,34,35,36]. Building upon this, more research is needed to illuminate what Black mothers need, desire, and prefer as supports for mental health, and the impact on maternal health and mental health outcomes.

## 3. Methods

### 3.1. Current Study

The aim of this study was to examine Black mothers’ maternal mental health needs, preferences, experiences, and barriers to support for mental health, and what healthcare professionals can do to be more supportive of their mental health. The research questions are: How do Black mothers make choices about accessing and using support for their mental health? What recommendations do Black mothers have for healthcare and other helping professionals to better support them?

### 3.2. Setting and Sample

The purposive sample of participants was recruited for this study from a midwestern metropolitan community from February 2020 to July 2020. Based on the precedence of the literature, participant recruitment occurred in natural spaces that Black mothers visit, such as childcare centers [32], Women, Infants, and Children (WIC) clinics, health clinics [27], home visiting programs [35], hair or nail salons [36], churches [37], and other community and online forums [36]. Taken together, a variety of recruitment strategies, specifically the use of flyers, presentations in community settings, and referrals from key knowledgeables in the community, facilitated access to the purposive sample. These practices are recommended in studies with racial and ethnic minority women [37,38]. Further, the planning and use of multiple strategies helped to facilitate recruitment and engagement in the study during a very difficult time.

Mothers who expressed interest in participating in the study were screened to determine eligibility for the study. The criteria included (a) self-identified as a Black or African American mother with a biological child under the age of 18 years in their care, (b) maternal age over 18 years, (c) self-reported mental health symptoms above the clinical cutoff of 16 and below 45 on the Center for Epidemiology Depression Scale (CES-D) [39] or self-identified mental health concerns at the time of the study pre-screening [32,33], and (d) resided in the county. The participants received $15 per hour for their participation through a reloadable debit card. The research was approved by the university’s institutional review board.

### 3.3. Data Collection

After the mothers agreed to participate and signed a consent form, they were engaged in an in-depth conversational semi-structured interview. The interviews were conducted by this researcher, who identifies as a Black mother from the same community. The interviews occurred in the setting the mothers preferred (e.g., virtually or in physical locations like their homes, parks, or coffee shops). The mothers were asked to participate in one interview and a follow-up conversation, if they were willing. The interviews lasted between 1 and 2.5 h. All the interviews were audio-recorded and transcribed verbatim. The transcripts were imported into NVivo 12 for data management and analysis.

### 3.4. Interview Guide

The interviews began with completing the consent form. After that process, there was a conversational style in the interviews, with carefully selected questions. With a slower pace to facilitate reflection, the interviews lasted between 1 and 2.5 h [40]. The questions in the interview were ordered to build rapport and focus on exploring the relationship between mental health symptoms, sources of support, and experiences (both positive and conflictual). The questions began with a conversation about meanings and experiences with social support and mental health. The guide included questions about the intersection of mental health and sources of support (informal, community, and formal mental health), types of support provided, and experiences sharing mental health with different sources of support. The participants were asked to describe supports for mental health and share experiences.

### 3.5. Reflexivity

My positionality as a Black, married woman, a mother of a young child, and mimi (stepmother) of two bonus children, with advanced education and living a somewhat middle-class experience as an infant mental health practitioner, more specifically, social worker and provider that supports the social, emotional, and mental health needs of perinatal people and families with young children birth to five from the same metropolitan context—shapes the way that I perceive and experience the world and, subsequently, how I approach this research. Even with the points of connection in mind between myself and the participants, I did not assume that I understood the context and experiences of the participants. However, instead of assuming that my personal life experiences, clinical work experience, scholarship, and research were invalid and should be excluded from this research, I avoided decontextualizing myself, my experiences, privileges, and disadvantages tied to this research by acknowledging it, explicating it, and considering the ways that this might influence this study [40,41]. I documented my reflections in reflexive memos and discussed them in peer debriefings with peers and advisors during the active research process and dissemination. My identities and experiences influenced a more nuanced understanding of Black maternal mental health and equity, sensitivity to participants’ and my own experiences, and the sharing of a fuller story through storytelling, with attention to the medium, such as artistic expression [42] and the mode of sharing.

### 3.6. Participants

Twelve Black women participated in this study. The participants’ ages varied from 18 to 40 years old. 50% of the participants identified their family status as single. The women were parenting from one child to three children under the age of 18. Nine of the women were pregnant and/or parenting a child in early childhood, from birth to 8 years old. In terms of the highest education level, half of the participants completed a master’s degree or higher, two graduated from college, three had some college or technical school, and one completed high school. Most of the participants were working full-time at the time of the study (n = 10) and had private insurance through their employers. Five of the mothers reported a history of mental illness or a mental health condition diagnosed by a health or mental health professional. Four participants presented with depressive symptoms in the normal range, four participants with mild or subclinical symptomology, and four participants with moderate to severe symptoms at the time of the interview. Table 1 will situate the findings in the context of both the mothers’ identity characteristics and some of their risk factors for poor mental health, more specifically, the identity characteristics (e.g., family status, number of children), and selected mental health risk factors (e.g., low income, unemployed) to help better understand each mother, individually, as well as understand the experiences of this group of mothers.

### 3.7. Data Analysis

Each interview was transcribed verbatim, and the coding process began [40]. While cleaning the transcripts, memoing, coding, and diagraming were practices used, and this allowed for gaining a clearer understanding of the individual’s experiences and the meaning of the units of data [40,43]. Concurrently, analyzing and collecting data is part of the iterative process. The data was reviewed repeatedly; data was defined and grouped in the coding process using open, focused, and theoretical coding. This comparative process was used to understand, describe, and explain a range of experiences, and the demographic characteristics of existing participants, and differences in family status, maternal experiences, and experiences with their mental health were examined. To analyze the data, a variety of strategies were used to rework the fragmented data into a coherent story, as well as answer the research questions. For example, the use of images and metaphors, walking in nature, morning reflections, and meditation were grounding and helped with meaning-making to bridge the gap between the coding and sense-making process. This was part of the theorizing process. To move towards the themes and describe categories with rich and thick descriptions, as outlined in this paper, the constant and comparative process and matrices were developed to think deeply about the phenomena, and describe the conditions, context, characteristics, and move towards the themes, clarity, and integration. Themes and quotes are included in this manuscript. The Consolidated Criteria for Reporting Qualitative Research Studies (COREQ) guided the reporting of methods and results in this paper [44].

## 4. Findings

Through the analysis, three core themes emerged related to ways that healthcare professionals can be helpful or supportive: expanding conceptions of support for mental health, preferences for support, and supporting mental health decision-making. Expanding conceptions of support for mental health is a theme describing the social relationships or people that Black mothers share their mental health concerns with, as well as the mental health-promoting activities or supportive actions. Preferences for support are the different preferences or desired characteristics of supports for mental health identified by the participants. The last theme supporting mental health decision-making reflects the knowledge and skills healthcare professionals can gain, and the actions that they can take to apply the insights shared by these Black mothers and become more helpful. The themes and illustrative quotes will be described briefly and outlined in the included tables.

### 4.1. Expanding Conceptualization of Supports for Mental Health

Participants identified a wide range of supports for mental health that include and go beyond formal mental health services. The participants highlight the vital role of informal and community-based resources (See Table 2). The types of support for mental health within their broadened conceptualization included self-care practices (e.g., journaling, self-talk, prayer, and cooking), informal support from family and friends, community supports (e.g., church groups), workplace supports (e.g., peer support, groups, employee assistance), and formal mental health providers (e.g., therapists and physicians). Many mothers described relying on personal coping mechanisms—journaling allowed emotional processing, while spiritual practices like prayer provided comfort, guidance, and strength. “You pray about it. That’s how you… some stuff, all you can do is pray about it because even talking to people don’t change it all the time,” explained Melanie, a single mother of three.

**Figure 1:**
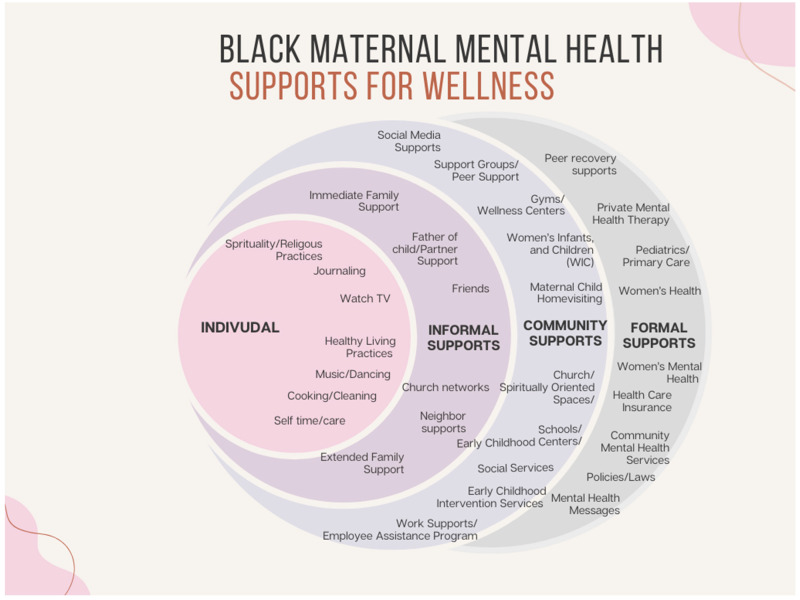
Supports for Black Maternal Mental Health and Wellness.

Connections and conversations about mental health were private, sensitive, and carefully chosen; participants often limited disclosure to a small group of supportive individuals, such as partners, mothers, close friends, or siblings. “If my therapist isn’t available, I talk to my husband. If my husband’s not available, I’ll call my mom. …I’m spiraling,” shared Remy, a married mother of two. Sharing certain aspects of one’s mental health experience, at certain times and spaces, was notable. Community spaces, especially churches and supportive workplaces, were important for emotional regulation and encouragement. “My boss at work… she’s like, ‘what’s wrong?’… Just be cool like this all the time, this would help with my anxiety and depression,” said Kesha, single mother of one. She illuminated how helpful it would be to encounter helpful individuals who can be trusted within the context of everyday life. Several of the mothers mentioned finding support for their mental health as they engaged in normal routines or activities within regularly visited spaces (e.g., home, church, child’s school, work, and health care settings). Further, it was notable that among the varied sources of support, some were seen as both helpful and stressful at times. Table 2 outlines the supports for mental health identified by the participants, along with illustrative quotes, and Figure 1 visualizes the broadened conceptualization of supports for mental health and wellness that are considered, used, and influencing Black maternal mental health and decision-making.

### 4.2. Preferences for Support

The varied experiences, both positive and negative, with different supports for mental health, appeared to intersect with the participant’s preferences. Participants expressed clear preferences regarding how to address their mental health needs or who they are or would talk to about mental health-related experiences, and the characteristics that they found helpful within their supports for their mental health (See Table 3). The participants had preferences for support among the different types of supports for mental health, with most indicating a preference for engaging in self-care or coping strategies, and with informal supports, namely their family member (sister, mother), partner or spouse, and close friends. Additionally, there were social identity characteristics that were preferred, or that the participants reported as important or helpful. These characteristics aligned with the participant’s lived experience, including race, gender, age, faith, and background. For example, Sharon, widowed mother of two reported, “I would like to talk to a woman… I’m a woman of color. I would like for her to be a little older… have a relationship with God,” while Remy, married mother of two described valuing being close in age to her therapist, cultural match, and technology use: “I like that she’s younger… she’s into pop culture. She knows what’s happening, so I don’t have to explain. She’s younger, so she’s more text-friendly. So just even the “Hey girl, I got your text. I’ll call you… is good enough for me.” Overall, the characteristics the participants highlighted appeared to contribute to their sense of safety and comfort engaging with different types of supports for mental health.

There were other characteristics or skills that were found through the participants’ experiences that were also noted as preferred, such as being nonjudgmental, patient, and a good listener. Lisa reported, “I definitely need someone who is patient… I need someone who’s extremely patient… I definitely need someone who I definitely feel safe with talking to…” Patience and active listening overtime are supportive actions that could build the sense of safety that Lisa shared was essential in who she shared mental health-related concerns with. Bonnie, a single mother of two children, also highlighted the importance of patience and listening, or the frustration when it is lacking. “Sometimes if they would be more of an active listener. Let me finish telling what I got to say, don’t cut me off. Don’t think you already know the answer to what I’m about to say.” Who the person is, is as important as how they are when supporting these mothers’ mental health needs.

Participants also reported valuing logistical accessibility and technological convenience. One mom reported that she chose a support for her mental health that she connected to through work, and there was a mobile option. “They had a couple of different options… it’s more mobile. I just like the services that it had, so through work basically,” Lisa, a single mother of one reflected. Several of the mothers highlighted numerous responsibilities, and having limited time being a barrier to engaging with supports for their mental health and engaging in self-care practices; therefore, easy access and a convenient way to engage also seemed to save time.

In addition to desiring an easy-to-navigate support for mental health, some of the participants elevated how they avoid potentially challenging, cumbersome, or negative experiences. Prior negative experiences, such as breaches of confidentiality, feeling judged, and feeling further depleted in the process of being supported, influenced future help-seeking and shaped preferences. One mom, Kesha, highlighted how sometimes she does not desire to engage with her close friend because of past negative experiences. “My best friend, she’s gone keep it real… opinions, …I don’t feel like hearing what you got to say right now… my prayer group is a little more open, not as judgmental.” Another mother, Margarit, a married mother of three children and a bonus child, highlighted experiencing feeling drained by care seeking with regard to a friend and a healthcare professional. “I have a friend that I call… when I call her, I’m already drained, it becomes more draining. It’s like I’d rather just drain one time than to have to try to find a little bit of fuel for her to drain that too,” Margarit reported. She also described a draining and negative experience with a healthcare professional. When describing an encounter, she said,

“I saw the nurse practitioner. It’s was just, “get me in the first appointment that you can.” I went in there and she said, “What’s going on?” I told her [about the rage and crying for days] and she was like, “Yeah, I think you got some hormonal imbalance.” She said, “birth control can help or we can put you on Zoloft” and I refused to be put on Zoloft… She didn’t ask, “What’s going on that is causing this?” Have you guys had domestic violence issues?” …

Here, it is notable that Margarit was rightfully unsatisfied with her care; it did not appear that the provider was listening or asking helpful questions, and the lack of consideration of context and approach was blaming. This was not uncommon; several of the mothers shared examples of negative experiences in the process of sharing their mental health experiences and being supported, and for most, this contributed to their preference and decision to engage in self-care, spirituality, and coping as a source of support for mental health. Many mothers actually engaged with multiple types of supports for mental health concurrently, and for some, it appeared to be intentional and a strategy accounting for the strengths and limitations of the different supports for mental health.

### 4.3. Supporting Mental Health Decision-Making

The participants shared insights and recommendations that can guide healthcare professionals towards being more supportive and helpful (Table 4). Increasing understanding about the needs, preferences, and experiences of Black mothers and gaining skills to be more helpful, were noted. Additionally, the participants shared recommended actions to be more supportive of Black mothers individually and collectively. Based on the participants’ experiences and recommendations, it would be important to understand how Black mothers describe supports for mental health, identify their preferences, and leverage their needs, preferences, and barriers to make decisions about supports for mental health. While examples have been shared to illuminate this, this quote from Amari, a single mother of three, illustrates how and why she makes certain decisions and how this has changed over time.

“I would say just some friendships that maybe are not as beneficial, someone was always just pulling from me and I would give, give, give… let me stick with this one person, my sister who I know will about, we can balance each other out just fine and there’s not that problem.”

Gaining awareness of the strengths and limitations of social relationships, as well as different types of services and supports, appears to be helpful in supporting Black mothers and their decision-making.

Another relevant factor to better understand is how emotional readiness and culturally shaped values and beliefs shape decision-making. Some participants reported feeling conflicted about help-seeking, identifying superwoman expectations or guilt about needing help. For example, “I try to keep it to myself as much as possible, because… I feel guilty, I guess, for not being able to manage it on my own,” Sasha, a married mother of one, confessed. Another mother, Sharon, a widowed mother of two, reflected on her decision-making process,

“and still, I find myself trying to just encourage my own self and not necessarily go to those outside sources. And I really don’t know why I haven’t sought to go to the outside sources, but I guess just in that time, I didn’t feel like it was about me. I felt like I had to make sure that they [her kids] were okay.”

These types of experiences illuminate the need for skills to listen for unrealistic expectations, guilt or shame, lack of prioritization of self, etc., and to build capacity to facilitate a culturally sensitive dialogue about this and the ways it contributes to engaging with supports for mental health (or not).

In addition to increasing knowledge and skills through training, participants also indicated a need for healthcare and helping professionals to have community collaborations, and, in particular, with Black providers. This would allow healthcare professionals to make referrals and engage in a warm handoff in accessing and using supports and services for their mental health needs. “Just knowing, ‘Hey, I’m going to be able to connect with someone who’s African American and it’s not going to be a struggle to find them’… that would be huge,” said Amari. The participants advocated for mental health life coaching, which could be a natural helper, like beauticians, nail techs, and community mental health workers, therapy, and peer supports or support groups tailored to Black mothers, “if there’s like one specifically for Black moms, that would be so helpful,” (Sasha, a married mother of one). Most of the participants indicated that they preferred options that were confidential, low-barrier, more convenient supports, such as a “talk line” for support, which could include just venting or prayer.

Additionally, participants highlighted a need for things to change within organizations and systems, specifically, transformative training, and enacting policy changes to promote accountability and positive experiences in care. Organizational changes—such as shadowing clinical encounters for quality assurance and developing culturally responsive programming—were proposed to create safer, more empowering healthcare experiences. Skyla, a single, pregnant mother of three young children, shared an important practical recommendation: “Walk through the appointments with them [providers] and see how they treat people, watch, and observe how they work.” This illuminates a willingness to experience discomfort when sharing more sensitive matters with more people, and to forgo privacy in some ways to ensure safety and quality care.

## 5. Discussion

The findings from this study illustrate how Black mothers navigate complex systems of support to promote their mental health. Participants described a broad spectrum of supports for mental health—self-care strategies, informal relationships and supportive actions, community resources, and formal mental health services—each with unique contributions and ways of influencing their mental health and limitations. These results expand the prevailing understanding of maternal mental health supports, centering the strengths and agency of Black mothers in their decision-making. These findings build upon the extant literature, as well as make unique contributions that can guide policy, practice, and research.

### 5.1. Expanding the Definition of Support for Mental Health

Existing literature has primarily focused on disparities in access to formal services and adverse health outcomes among Black mothers, often overlooking the creative and resilient ways they seek support in their communities [5,16]. This study builds upon the literature demonstrating that Black mothers frequently prioritize informal and community-based resources, such as family, friends, church groups, and workplace relationships, for emotional and practical help. Such supports, when aligned by identities, offer safety, understanding, and empowerment—essential qualities often absent from formal settings [28,34]. By recognizing and validating these wider networks or systems of support and care, and positioning supports for Black maternal mental health within the proposed socio-ecological frame [16], healthcare professionals can develop more responsive models for care and have more helpful conversations about the options. Studies have shown that community-based supports, such as mindfulness stress reduction groups, support groups, and doulas and community mental health worker interventions, can help reduce mental health symptoms and access services and supports [3,29]. Contrary to the disjointed existing literature documenting the role and impact of informal, community, and formal mental health supports separately, these findings provide insights that indicate Black mothers are exploring and using a variety of supports for mental health and concurrently to meet their needs.

### 5.2. Preferences and Decision-Making

Given that very few studies capture preferences for informal, community, or formal mental health support in isolation among Black mothers, this study’s findings add uniquely to the literature. This study noted that Black mothers’ preferences are deeply shaped by prior experiences, cultural backgrounds, accessibility, and personal values and beliefs. Racialized stress and worry have been noted to contribute to preferences for informal and community care [34]. There were certain identifying characteristics that were preferred, such as race, faith, age, and gender. This aligns with prior studies that have indicated the necessity for Black women to experience racially concordant care to enhance safety, trust, and quality of care [27,28,41]. According to Murrell & Fleury (2024) [34], social safety is critical to facilitating positive experiences and better outcomes, and for Black mothers this is defined as “a process of feeling understood, respected, cared for, and in control in perinatal health care settings that make space, care for, and recognize strengths thereby cultivating safety and empowerment.” ([34], p. 768). The findings, highlighting the skills needed such as being nonjudgmental, patient, and attuned to their lived experiences, are ideal in support of their mental health. As in prior research, social safety is a key driver of preference for mental health supports, and more specifically, the preference for self as a support for mental health and engaging spirituality, along with other coping strategies reduce stress and addresses mental health needs. These findings suggest that healthcare systems and providers must prioritize social safety to improve engagement and outcomes for Black mothers.

Negative clinical or social interactions—such as judgmental attitudes, lack of cultural understanding, or dismissive treatment—were identified by participants as longstanding barriers. As in the recent literature, these experiences undermine social safety and perpetuate mistrust [27,33,45,46]. Studies do highlight that there are gaps in education and training, and that training could be an intervention to increase knowledge, skills, and the quality of care [28]. Additionally, future research could explore the impact of training for healthcare and helping professionals, as well as the extent to which negative experiences with healthcare providers contribute to their preferences for informal and community care and mental health decision-making. Further, future research could also explore how efforts to make social safety a core metric for quality improvement, staff training, and organizational reform.

### 5.3. Implications for Practice, Policy, and Research

The findings in this study contribute to the literature and there are several actionable directions for improving care:

- Healthcare organizations should invest in training on cultural humility and bias interruption, and build staff capacity for active listening and nonjudgmental support during shared decision-making about mental health. Establishing a compensated advisory board comprising Black mothers would provide critical ongoing insights and guidance for quality improvement.
- Healthcare systems must increase the representation of Black providers, foster meaningful and reciprocal partnerships with Black-led and women-led community organizations, and offer tailored mental health conversations and resource navigation services for Black mothers.
- Programs should create and promote support groups and confidential “talk lines” specifically designed with and for Black mothers, reducing barriers to access and enhancing psychological safety.
- Universal behavioral health screening policies and practices must be examined and reimagined, centering the lived experiences and guidance of Black women and mothers to become more helpful in facilitating safer, more culturally responsive screening experiences. There is an imperative to identify concerns early and intervene for Black mothers because of the disproportionate risk of experiencing maternal mental health concerns, intimate partner violence, substance use, and negative social determinants that influence the likelihood of experiencing more severe chronic and debilitating maternal mental health conditions and causing maternal death.
- Decision makers should implement reforms for greater accountability in clinical settings, including shadowing clinical encounters and systematically monitoring care quality for Black mothers. User-friendly avenues for reporting concerns, negative experiences, or discrimination should also be established and visible.
- Future research should be led by Black mothers using community-based participatory methods to examine how healthcare providers can best support Black mothers’ access and use of mental health supports. Research should examine similar questions with a larger sample in varied geographic and community contexts.
- Researchers should further investigate the effects of accessing and using multiple types of mental health supports (informal, community-based, and formal), with particular attention to those that are less studied or nondominant ways of supporting mental health needs. It would also be valuable to explore the outcomes associated with concurrent use of these different support types.
- Additional theoretical development regarding the relationship between preferences, experience, and decision-making is warranted, as is further evaluation of the actions, interventions, or programs proposed by participants to be more supportive of their mental health.

### 5.4. Strengths of the Study

This study offers several notable strengths. It centers the voices and lived experiences of Black mothers, elevating their definitions, preferences, and decision-making processes regarding supports for mental health in ways rarely documented in the literature. By employing in-depth interviews, the research surfaces nuanced understandings of the concurrent use of informal, community, and formal supports. The study had a small sample with rich, thick data, and findings could be transferable among Black mothers within other regions. The study’s focus on actionable, community-driven recommendations provides practical guidance for advancing maternal mental health equity within healthcare systems and organizations. Additionally, as with many qualitative studies, some findings may be influenced by the researcher positionality and participants’ willingness to share their experiences, and the racial congruence present in this study may have enhanced the experience and felt sense of safety [34,41].

Rigor in this qualitative study was important, and achieved through the methods rooted in established criteria for trustworthiness—credibility, dependability, confirmability, and transferability [40]. Strategies to promote trustworthiness included in-depth, conversational, semi-structured interviews, constant comparative analysis, memoing, and ongoing member checking with participants to ensure that interpretations accurately reflected their experiences [40]. The study maintained credibility by engaging participants in the validation of findings, employing a reflexive journal to document methodological decisions and researcher perspectives, and debriefing. Analytical rigor was further enhanced through iterative coding and diagramming, audit trails, and triangulation across data sources and analytic activities. Collectively, these efforts ensured that the results were firmly grounded in participants’ voices, and this demonstrated resonance and originality, and contributed usefully to the understanding of Black maternal mental health supports and what healthcare professionals can do to support their mental health.

### 5.5. Limitations of the Study

Despite the strengths, several limitations must be considered. A clearer understanding and theoretical saturation of the categories was approached, yet not complete. Due to study feasibility issues, specifically to meet the study timeline and funding for the project, the sample size was relatively small, and given the diversity among the Black mothers that participated—with variation in age, family status, socioeconomic status, and mental health histories, this limits the breadth of perspectives that could have been captured and the generalizability of the findings. Most of the participants resided in one midwestern metropolitan area, and despite the efforts to recruit participants with various socioeconomic statuses and family statuses, there were fewer participants who were experiencing low-income conditions. Qualitative researchers contend that when conducting research with heterogeneous samples, larger sample sizes are needed to fully understand the phenomena in ways that facilitate complex and comprehensive findings. Another related limitation is that the study took place in part during the global pandemic COVID-19; therefore, there are likely perspectives missing due to time, technology, mistrust, capacity, and experiencing health challenges, etc. While generalizability is desired within quantitative research, it is not a major evaluative criterion of qualitative research; however, it is important to be transparent about sample size limitations and acknowledge the lived experiences represented (or not) in this study and the intersecting identities of participants within the group. In qualitative research, transferability is important, and the insightful and detailed descriptions provided, heterogeneity of the participants, and documented limitations, and this enhances the capacity of the reader to assess the applicability of the findings to different settings with Black mothers.

## 6. Conclusions: A Call to Action

Aligned with the aims of the special issue, advancing mental health equity for marginalized communities requires addressing both social determinants and structural barriers to care, fostering culturally responsive strategies, and elevating the agency, strengths, and preferences of Black mothers. Organizational reforms—such as increasing provider diversity, enacting quality assurance practices, and facilitating community partnerships—are critical for shifting the paradigm from deficit-oriented to strengths-based models of care. What you can do has been outlined, and with that, what will you do? Future research and policy should prioritize mental health equity, and in centering Black mothers’ lived experiences, preferences, and resilience, we will accelerate progress in maternal mental health equity, which will benefit Black mothers, their children, families, communities, and our society.

## Figures and Tables

**Table 1:** Demographic Characteristics (n = 12).

Characteristics	Count	Percentage %
**Age (in Years)**		
18–23	1	8
23–28	0	0
29–34	5	42
35–40	6	50
**Family Status**		
Single	6	50
Living with partner	1	8
Married	4	33
Widowed	1	8
**Number of Children**		
1	4	33
2	4	33
3+	4	33
**Pregnant or Parenting Child 0 to 8**		
No	3	25
Yes	9	75
**Highest Level of Education**		
High school graduate/GED	1	8
Some college or technical school	3	25
4-year college graduate	2	17
Post graduate degree	6	50
**Work Status**		
Employed (full-time)	10	84
Not employed	1	8
Not employed looking for work	1	8
**Household Annual Income**		
Between $10,000–20,000	3	25
Between $20,001–40,000	2	17
Above $40,000	7	58
**Health Insurance Status**		
Medicaid/Medicare	2	17
Private Insurance Provider	9	75
Other	1	8
**History of Mental Illness**		
No	7	58
Yes	5	42
**CES-D Score**		
Normal symptoms: 0–15	4	33
Mild/Moderate symptoms: 16–23	4	33
Moderate Symptoms: 24–30	2	17
Moderate/Severe Symptoms: 31–60	2	17

**Table 2:** Supports for Mental Health.

Theme	Description	Dimensions	Illustrative Quotes
Self, Self-Help, Coping	Individual mental health-promoting activities and internal strategies	Journaling, self-talk, spirituality, cooking	“I try to journal before I run to talk to someone else to help me… kind of process it.” (Sasha, married mother of one) “Last night I think I cried for like three minutes, but they were really coming out, those tears… then I just remembered oh I got a whole nother life on me. Let me stop… I just focused on the baby.” (Amber, a pregnant and married mother of two)
Informal Supports (Family, Friends)	Emotional and practical supports from close relationships	Partner, family, close friends,	“If my therapist not available, I talk to my husband. If my husband’s not available, I’ll call my mom… I’m spiraling.” (Remy, married mother of two) “I feel like with the stressors in my mental health, if I talk to anyone, it’s usually my close friends and I have probably like two really extremely supportive friends who I can just kind of be open with and don’t feel like I’m being judged for it.” (Sasha, married mother of one)
Community Supports	Support from non-family, broader networks	Church groups, workplace, prayer group	“My boss at work… Just be cool like this all the time, this would help with my anxiety and depression.” (Kesha, single mother of one) “recently, I’ve kind of hit my tipping point … I tend be an I got it person. I had it until apparently I didn’t, but I didn’t realize I didn’t have it until I actually went to my pastor. I broke down crying.” (Margarit, married mother of three children and a bonus child)
Formal Services and supports	Professional support within health and mental health sector	Mental health screening, therapy, medication, case management, health care	“I see a therapist because I have anxiety. I actually got it connected through my job… all my meetings are via Zoom.” (Lisa, a single mother of one) “[My doctor] She mentioned to me not only do you need to get this medication but you also need to talk to a therapist.” (Kesha, single mother of one)

**Table 3:** Preferences for Support.

Theme	Description	Dimensions	Illustrative Quote
Types of support	Different types of support preferred	Identifying different types of support that are preferred, self, informal, community, formal mental health	“If there was some type of… parent support groups specifically for black moms… that would be so helpful.” (Brooke, single mother of one) “my best friend, she’s gone keep it real, the opinions, okay, I don’t feel like hearing what you got to say right now… Whereas I feel like my prayer group is a little bit more open where they’re not as judgmental as she can be some time” (Kesha, single mother of one)
Preferred Characteristics	Qualities desired in support persons	Preferring certain identities and lived experiences, ways of being nonjudgmental, patient, and safe	“I would like to talk to a woman… I’m a woman of color. I would like for her to be a little older… have a relationship with God.” (Sharon, widowed mother of two) “be more of an active listener. Let me finish telling what I got to say, don’t cut me off. Don’t think you already know the answer to what I’m about to say.” (Bonnie, single mother of two children)
Logistical Accessibility	Value placed on convenience, technological capability	Identifying convenient and easy-to-use mobile services, work-based access, flexibility	“They had a couple of different options… it’s more mobile. I just like the services that it had, so through work basically.” (Lisa, a single mother of one) “Because she’s younger, she’s technologically savvy… so she’s more accessible, which I appreciate.” (Remy, married mother of two)
Previous Negative Experiences	Identified negative experiences shaping thoughts about supports for mental health and preferences	Negative experiences such as confidentiality breaches, judgmental and dismissive encounters, and other unhelpful, stressful experiences	“I tried journaling, but then my husband found my journal… You know what? I’m done.” (Sasha, married mother of one) “I have a friend that when I call her, I’m already drained, it becomes more draining. It’s like I’d rather just drain one time than to have to try to find a little bit of fuel for her to drain that too…” (Margarit, married mother of three children and a bonus child)

**Table 4:** Mental Health Decision-Making.

Theme	Description	Dimensions	Illustrative Quotes
Building knowledge and skills	Knowledge, skills, attitudes, and training of the healthcare professionals can be improved to provide quality, culturally responsive care	Awareness of supports for mental health, preferences, and cultural responsiveness	“If they would be more of an active listener. Let me finish telling what I got to say, don’t cut me off.” (Brooke, single mother of one) “Be aware of your response… there is a huge need for more culture training.” (Bonnie, single mother of two children)
Building knowledge and skills	Knowledge, skills, attitudes, and training of the healthcare professionals can be improved to provide quality, culturally responsive care	Capacity to identify emotional readiness, personal barriers and support autonomy	“I try to keep it to myself as much as possible… I feel guilty… for not being able to manage it on my own.” (Brooke, single mother of one) “I find myself trying to just encourage my own self and not necessarily go to those outside sources. And I really don’t know why I haven’t sought to go to the outside sources, but I guess just in that time, I didn’t feel like it was about me. I felt like I had to make sure that they were okay.” (Sharon, widowed mother of two)
Intentional Action for Equity	Specific actions recommended that can address inequities and promote maternal mental health equity	Building strong community collaborations, especially with Black providers and capacity to facilitate conversations about options	“Just knowing… I’m going to be able to connect with someone who’s African American and it’s not going to be a struggle to find them… that would be huge.” (Amari, single mother of three) “If there’s like one specifically for black moms, that would be so helpful.” (Sasha, married mother of one)
Intentional Action for Equity	Specific actions recommended that can address inequities and promote maternal mental health equity	Developing and identifying supports that provide access to emotional support, peer support, and resource navigation	“I think they should make like a hotline… If you want them to pray for you, they can pray for you.” (Melanie, single mother of three) “There’s a lot of resources out there that people might not just might not know how to access or they don’t know if there’s… I don’t know how to access it.” (Amari, single mother of three)
Intentional Action for Equity	Specific actions recommended that can address inequities and promote maternal mental health equity	Promoting organizational policy change for action and accountability to protect Black mothers	“Walk through the appointments with them [providers] and see how they treat people, watch, and observe how they work.” (Skyla, single, pregnant mother of 3 young children)

## Data Availability

The qualitative data supporting the conclusions of this article are not publicly available due to the sensitive nature of the information and to protect participant confidentiality and privacy. The data contain personal mental health experiences and detailed narratives that could potentially identify participants despite the use of pseudonyms. Access to anonymized excerpts may be available from the corresponding author upon reasonable request and with appropriate ethical approval, subject to institutional review board guidelines and participant consent provisions.
